# *Salmonella enterica* in farm environments in the Ashanti Region of Ghana

**DOI:** 10.1186/s12866-023-03121-3

**Published:** 2023-11-29

**Authors:** Linda Aurelia Ofori, Dennis Fosu, Seth Ofori, Charity Wiafe Akenten, Antje Flieger, Sandra Simon, Anna Jaeger, Maike Lamshöft, Juergen May, Kwasi Obiri-Danso, Richard Phillips, Daniel Haile Chercos, Ellis Kobina Paintsil, Denise Dekker

**Affiliations:** 1 Department of Theoretical and Applied Biology, Kwame Nkrumah University of Science and Technology (KNUST), 039-5028 Kumasi, Ghana; 2 Kumasi Centre for Collaborative Research in Tropical Medicine (KCCR), South-End, Asuogya Road, Kumasi, Ghana; 3 Unit Enteropathogenic Bacteria and Legionella, National Reference Center for Salmonella and Other Bacterial Enteric Pathogens, Robert-Koch-Institute (RKI), Burgstr. 37, 38855 Wernigerode, Germany; 4 Department Infectious Disease Epidemiology, Bernhard Nocht Institute for Tropical Medicine (BNITM), Bernhard-Nocht-Str. 74, 20359 Hamburg, Germany; 5 German Centre for Infection Research (DZIF), Hamburg-Lübeck-Borstel-Riems, 38124 Braunschweig, Germany; 6 Tropical Medicine II, University Medical Centre Hamburg-Eppendorf (UKE), Hamburg, Germany; 7 Department Implementation Research, One Health Bacteriology Research Group, Bernhard Nocht Institute for Tropical Medicine (BNITM), Bernhard-Nocht-Str. 74, 20359 Hamburg, Germany

**Keywords:** *Salmonella enterica*, Environmental reservoirs, Antibiotic resistance, Farms, Rural Ghana

## Abstract

**Background:**

*Salmonella enterica* are important foodborne pathogens and the third leading cause of death among diarrheal infections worldwide. This cross-sectional study investigated the frequency of antibiotic-resistant *Salmonella enterica* in commercial and smallholder farm environments in the Ashanti Region of Ghana. A total of 1490 environmental samples, comprising 800 (53.7%) soil (from poultry, pigs, sheep, goats and cattle farms), 409 (27.4%) pooled poultry fecal and 281 (18.9%) dust (from poultry farms) samples, were collected from 30 commercial and 64 smallholder farms. All samples were processed using standard culture methods. Isolates were identified by biochemical methods and confirmed using the VITEK 2 System. Antibiotic susceptibility testing was carried out by disk diffusion following the EUCAST guidelines. Serotyping was performed using the Kauffman White Le Minor Scheme.

**Results:**

The overall *Salmonella* frequency was 6.0% (n/N = 90/1490); the frequency varied according to the type of sample collected and included: 8.9% for dust (n/N = 25/281), 6.5% for soil (n/N = 52/800) and 3.2% for pooled poultry fecal samples (n/N = 13/409). *Salmonella* was also recovered from commercial farm environments (8.6%, n/N = 68/793) than from smallholder farms (3.2%, n/N = 22/697) (PR = 2.7, CI: 1.7 – 4.4). Thirty-four different *Salmonella* serovars were identified, the two most common being Rubislaw (27.8%, n/N = 25/90) and Tamale (12.2%, n/N = 11/90). Serovar diversity was highest in strains from soil samples (70.6%, n/N = 24/34) compared to those found in the dust (35.2%, n/N = 12/34) and in fecal samples (29.4%, n/N = 10/34). *Salmonella* frequency was much higher in the rainy season (8.4%, n/N = 85/1007) than in the dry season (1.0%, n/N = 5/483) (PR = 8.4, 95% CI: 3.3 – 20.0). Approximately 14.4% (n/N = 13/90) of the isolates were resistant to at least one of the tested antimicrobials, with 84.6% (n/N = 11/13) being resistant to multiple antibiotics. All *Salmonella* Kentucky (*n* = 5) were resistant to ciprofloxacin.

**Conclusion:**

This study showed that farm environments represent an important reservoir for antibiotic-resistant *Salmonella*, which warrants monitoring and good husbandry practices, especially in commercial farms during the rainy season, to control the spread of this pathogen.

## Background

Recent data suggest that non-typhoidal *Salmonella* invasive disease causes between 46,400 and 123,000 annual deaths worldwide, mostly in sub-Saharan Africa [1]. So far, more than 2,600 *Salmonella* serovars have been described. *Salmonella* transmission in humans is typically of zoonotic origin, and the majority of infections in both humans and animals are caused by subspecies 1 of *Salmonella enterica* [2]. Nevertheless, *Salmonella enterica* has not been widely studied in sub-Saharan Africa [3]. Even though anthroponotic transmission has been suggested, it is yet to be confirmed [4].

Antimicrobial resistance (AMR) leading to difficult-to-treat *Salmonella* infections in humans is a global health concern [5]. Multidrug-resistant (MDR) *Salmonella* and emerging fluoroquinolone resistance are rising worldwide [6]. In Ghana, resistant *Salmonell*a have been reported from humans [7, 8], farm animals [9, 10], water sources [11], fresh milk [12] and vegetables [13]. Despite Ghana having a national action plan for antimicrobial use [14], poultry and livestock farmers continue to overuse antimicrobials such as oxytetracycline, neomycin and tylosin for growth promotion, prophylaxis and infection treatment [15], fostering the development of AMR.

*Salmonella* are highly adaptive bacteria that can persist in the environment for prolonged periods [16]. The presence of *Salmonella* in soil may originate from fecal droppings, organic fertilizers or dust from farms [17]; hence environmental niches such as soil and dust might present possible transmission reservoirs for infections. So far, only a few studies have been conducted on *Salmonella* in soil and dust, and data for Africa is limited [18, 19]. The majority of *Salmonella* studies conducted in Ghana focused on commercial farm animals [9, 10], slaughterhouses [20, 21] and markets [13, 22]. In this study, we examined the frequency and antibiotic resistance of *Salmonella enterica* isolated from environmental samples (dust and soil) and animal feces collected from commercial and smallholder farm environments in two communities in the Ashanti Region of Ghana.

## Results

### *Salmonella* frequencies in environmental samples from rural and semi-urban communities

A total of 1490 environmental samples, comprising 800 (53.7%) soil, 409 (27.4%) pooled fecal and 281 (18.9%) samples from dust, were collected from 30 commercial and 64 smallholder farms in the rural Agogo and semi-urban Ejisu communities of Ghana. The overall *Salmonella* frequency was 6.0% (n/N = 90/1490). Frequencies from the two study sites: rural (4.9%, n/N = 37/750) and semi-urban (7.2%, n/N = 53/740), were similar (PR = 0.7, 95% CI: 0.5 – 1.0). Overall *Salmonella* frequencies varied according to the type of sample collected: 8.9% (n/N = 25/281) for dust, 6.5% (n/N = 52/800) for soil and 3.2% (n/N = 13/409) for fecal samples. The highest frequency recorded by commercial farms was observed in samples from dust (20.8%, n/N = 15/72) followed by soil (13.1%, n/N = 34/260) samples. Out of the 40 smallholders and 15 commercial farms sampled from rural Agogo, 12 (30%) and 6 (40%) were positive for *Salmonella*, respectively. Also, in the semi-urban community, more commercial farms (86.7%, n/N = 13/15) were positive for *Salmonella* than smallholder farms (8.3%, n/N = 2/24) (PR = 10.4, 95% CI: 2.7 – 39.8). Table 1 details *Salmonella* frequencies detected from the various sample types and study sites.

**Table 1:** *Salmonella* frequencies detected from various sample types from rural (Agogo) and semi-urban (Ejisu) communities

Sample type	Rural, % (n/N)		Semi-urban, % (n/N)		Total, % (n/N)
	**Commercial**	**Smallholder**	**Commercial**	**Smallholder**	
Soil	4.3 (8/186)	4.2 (9/214)	13.1 (34/260)	0.7 (1/140)	6.5 (52/800)
Fecal	1.0 (1/104)	9.5 (10/105)	2.0 (2/100)	0 (0/100)	3.2 (13/409)
Dust	11.3 (8 /71)	1.4 (1/70)	20.8 (15/72)	1.5 (1/68)	8.9 (25/281)
**Total**	**4.7 (17/361)**	**5.1 (20/389)**	**11.8 (51/432)**	**0.6 (2/308)**	**6.0 (90/1490)**

*n* positive sample, *N* total sample size

### Factors associated with *Salmonella* frequencies

Table 2 shows a Poisson regression analysis of possible factors associated with *Salmonella* frequencies. From the adjusted prevalence ratios, more *Salmonella* were recovered from commercial farms (8.6%, n/N = 68/793) than from smallholder farms (3.2%, n/N = 22/697) (PR = 2.6, CI: 1.6 – 4.3). Also, *Salmonella* was 2.7 (95% CI: 1.4 – 5.5) and 1.9 (95% CI: 1.1 – 3.6) times more likely to be isolated from dust and soil than from fecal samples. In the farm environment, *Salmonella* isolation rates in soil within ≤ 5 m around the pen and > 5 m away from the pen were similar (PR = 1.3, 95% CI: 0.6 – 2.4).

**Table 2:** Poisson regression analysis of possible factors associated with *Salmonella* frequency

Variable	Crude ratios PR (95% CI)	Adjusted ratios PR (95% CI)
**All *Salmonella* isolates**		
Commercial vs. smallholder farms	2.7 (1.7 – 4.4)	**2.6 (1.6 – 4.3)**
Ejisu (semi-urban) vs. Agogo (rural)	1.4 (0.9 – 2.2)	1.3 (0.8 – 2.0)
Dust vs. fecal	2.8 (1.4 – 5.6)	**2.7 (1.4 – 5.5)**
Dust vs. soil	1.3 (0.8 – 2.1)	1.4 (0.8 – 2.3)
Soil vs fecal	2.0 (1.1 – 3.9)	**1.9 (1.1 – 3.6)**
**Soil* Salmonella* isolates**		
Poultry vs. Other livestock	0.5 (0.3 – 0.8)	**0.5 (0.3 – 0.9)**
≤ 5 m near pen vs. > 5 m away from pen	1.4 (0.8 – 2.4)	1.3 (0.6 – 2.4)

### *Salmonella* serovar distribution

All *Salmonella* isolated from environmental samples belonged to the *Salmonella enterica* species. The vast majority (96.7%, n/N = 87/90) belonged to the subspecies *enterica*, 2.2% (n/N = 2/90) belonged to the subspecies *diarizonae* and 1.1% (n/N = 1/90) were subspecies *salamae* (Table 3). The *diarizonae* and *salamae* subspecies were only found in the soil. A total of 34 different *Salmonella* serovars were identified in this study. Serovar diversity was highest in strains from soil samples (70.6%, n/N = 24/34) compared to those found in the dust (35.2%, n/N = 12/34) and in fecal samples (29.4%, n/N = 10/34). Also, the serovar diversity from smallholder farms (77.2%, n/N = 17/22) was broader than what was found in commercial farms (29.4%, n/N = 20/68) (PR = 2.3, 95% CI: 1.6 – 3.2). Overall, the two most common serovars were Rubislaw (27.8%, n/N = 25/90) and Tamale (12.2%, n/N = 11/90). Most serovar Tamale (72.7%, n/N = 8/11) and Rubislaw (68.0%, n/N = 17/25) were identified from dust and soil, respectively (Table 3).

**Table 3:** Distribution of *Salmonella* serovars isolated from environmental samples

*subsp.*	serovar	Soil, *N* = 52 % (n)	Dust, *N* = 25 % (n)	Fecal, *N* = 13 % (n)	Total, *N* = 90 % (n)
*enterica*	Rubislaw	32.7 (17)	24.0 (6)	15.4 (2)	27.8 (25)
	Tamale	1.9 (1)	32.0 (8)	15.4 (2)	12.2 (11)
	Kentucky	5.8 (3)	8.0 (2)	-	5.6 (5)
	Bochum	5.8 (3)	4.0 (1)	-	4.4 (4)
	Yovokome	3.8 (2)	-	15.4 (2)	4.4 (4)
	Agona	5.8 (3)	-	-	3.3 (3)
	Reading	3.8 (2)	-	7.7 (1)	3.3 (3)
	Westphalia	3.8 (2)	-	-	2.2 (2)
	Montevideo	3.8 (2)	-	-	2.2 (2)
	Chester	1.9 (1)	4.0 (1)	-	2.2 (2)
	Epinay	1.9 (1)	4.0 (1)	-	2.2 (2)
	Ilala	1.9 (1)	4.0 (1)	-	2.2 (2)
	Agama	1.9 (1)	-	-	1.1 (1)
	Alachua	1.9 (1)	-	-	1.1 (1)
	Duisburg	1.9 (1)	-	-	1.1 (1)
	Give	1.9 (1)	-	-	1.1 (1)
	Honelis	1.9 (1)	-	-	1.1 (1)
	Poona	1.9 (1)	-	-	1.1 (1)
	Mundonobo	1.9 (1)	-	-	1.1 (1)
	Saphra	1.9 (1)	-	-	1.1 (1)
	Serologically Rough	1.9 (1)	-	-	1.1 (1)
	Typhimurium	-	4.0 (1)	-	1.1 (1)
	Durban	-	4.0 (1)	-	1.1 (1)
	Elisabethville	-	4.0 (1)	-	1.1 (1)
	Redhill	-	4.0 (1)	-	1.1 (1)
	Konongo	-	4.0 (1)	-	1.1 (1)
	Aschersleben	-	-	7.7 (1)	1.1 (1)
	Gaminara	-	-	7.7 (1)	1.1 (1)
	Wagenia	-	-	7.7 (1)	1.1 (1)
	Wien	-	-	7.7 (1)	1.1 (1)
	Lexington	-	-	7.7 (1)	1.1 (1)
	Unidentified	3.8 (2)	-	7.7 (1)	3.3 (3)
*diarizonae*	Ssp. IIIb	3.8 (2)	-	-	2.2 (2)
*salamae*	Ssp. II	1.9 (1)	-	-	1.1 (1)
**Total**		**100 (52)**	**100 (25)**	**100 (13)**	**100 (90)**

### Seasonal variation of *Salmonella* frequency

**Fig. 1:**
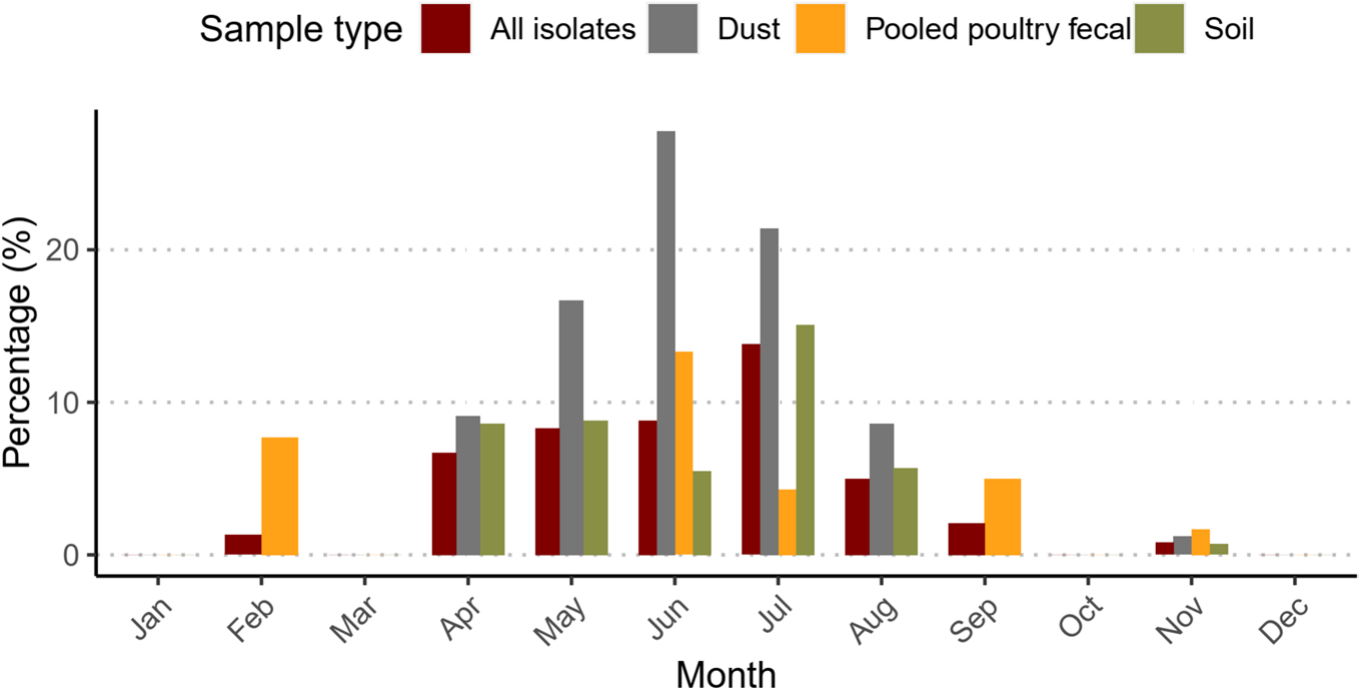
Seasonal frequencies of *Salmonella* isolated from dust, pooled fecal and soil samples collected from farm environments

Figure 1 shows the monthly isolation rate of *Salmonella* from dust, fecal and soil during the sampling period. In January, March, October and December, no *Salmonella* were isolated. The highest *Salmonella* frequency from dust (23.6%, n/N = 17/74), soil (12.3%, n/N = 15/122) and fecal (7.8%, n/N = 9/115) samples were recorded between June and July. Furthermore, the overall *Salmonella* frequency was much higher in the rainy season (8.4%, n/N = 85/1007) than in the dry season (1.0%, n/N = 5/483) (PR = 8.4, 95% CI: 3.3 – 20.0).

### Antimicrobial resistance

Among all the antibiotics tested, the highest rate of resistance was to ciprofloxacin (12.2%, n/N = 11/90), whereas all *Salmonella* were susceptible to cefotaxime, cefoxitin, ceftazidime and meropenem. In terms of serovars and associated resistance, all *Salmonella* Kentucky (*n* = 5) were resistant to ciprofloxacin. Ciprofloxacin resistance was also found in the serovars Chester (18.1%, n/N = 2/11), Ilala (18.1%, n/N = 2/11), Montevideo (9.0%, n/N = 1/11), and Epinay (9.0%, n/N = 1/11). None of the strains was MDR (resistant to chloramphenicol, ampicillin, and trimethoprim-sulfamethoxazole), but 14.4% (n/N = 13/90) of the *Salmonella* were resistant to at least one tested antibiotic, and 12.2% (n/N = 11/90) were resistant to multiple antibiotics. All of the 11 multiple antibiotic resistant isolates were either from the soil (54.5%, n/N = 6/11) or dust (45.4%, n/N = 5/11). Multiple antibiotic resistance was seen in the serovars Epinay, Montevideo, Chester, Ilala, and Kentucky. Table 4 further shows the observed antibiotic resistance in *Salmonella* serovar isolated from the different sample types.

**Table 4:** Antibiotic resistance patterns in different *Salmonella* serovars isolated from environmental samples

Sample type	Serovar	Resistance, % (n/N)			
		Ampicillin	Ciprofloxacin	Trimethoprim-sulfamethoxazole	Chloramphenicol
Soil	Kentucky	100 (3/3)	100 (3/3)	33.3 (1/3)	0 (0/3)
Soil	Montevideo	50 (1/2)	50 (1/2)	0 (0/2)	0 (0/2)
Soil	Chester	0 (0/1)	100 (1/1)	100 (1/1)	0 (0/1)
Soil	Ilala	0 (0/1)	100 (1/1)	100 (1/1)	0 (0/1)
Dust	Kentucky	100 (2/2)	100 (2/2)	50 (1/2)	0 (0/1)
Dust	Epinay	100 (1/1)	100 (1/1)	100 (1/1)	0 (0/1)
Dust	Chester	0 (0/1)	100 (1/1)	100 (1/1)	0 (0/1)
Dust	Ilala	0 (0/1)	100 (1/1)	100 (1/1)	0 (0/1)
Fecal	Lexington	0 (0/1)	0 (0/1)	0 (0/1)	100 (1/1)

## Discussion

This cross-sectional study investigated the frequency and antimicrobial resistance of *Salmonella enterica* isolated from soil, dust and fecal samples collected from commercial and smallholder farms in two communities in the Ashanti Region of Ghana. The overall *Salmonella* frequency observed in this study was 6%. This raises concerns for public health, particularly for workers and individuals residing near these farms, as *Salmonella* is a zoonotic pathogen with the potential to cause foodborne illness in humans. The current *Salmonella* frequency detected is similar to what was reported from studies conducted in Nairobi (2.6–5.9%) [23] and Nigeria (10%) [24]. However, it is far less than the 44% reported by a similar study from commercial poultry farms and markets in Ghana [9]. This disparity could be due to the differences in the types of environmental samples analyzed and the geographical locations. In commercial farms, we observed the highest *Salmonella* frequency in the dust. In agreement with the current findings, several studies have also associated dust generated in farms with *Salmonella* transmission in farm animals and sporadic human outbreaks [25, 26].

In humans, *S*. Enteritidis and *S*. Typhimurium have been reported as the most common serovars isolated from clinical samples [27]. Our study observed a very low prevalence of *S*. Typhimurium and no* S*. Enteritidis. This could be attributed to the fact that *S*. Typhimurium is more frequently associated with human socio-demographic features rather than environmental reservoirs [28]. Interestingly, our study found no *S.* Enteritidis, while a similar previous study conducted in Kumasi, Ghana, reported a 10.6% prevalence of this serovar [9]. This suggests the need for further research to confirm whether the *Salmonella* serovar distribution in the farm environments in Ghana is changing. Nonetheless, we identified other *Salmonella enterica* serovars, potentially capable of causing human infections but known to inhabit various environmental sources, including soil, water, plants, and animals. The current study observed a diverse serovar distribution in the environment, mostly isolated from soil and dust. This observation is not unusual since earlier findings have reported diverse serovars in environmental samples [29, 30]. The most common serovars identified in this study were *S*. Rubislaw and *S*. Tamale. In agreement with our findings, *S.* Rubislaw has been recovered in Ghana from dust and poultry feces [9] and drinking water sources [31]. In Australia, the first recorded instance of *Salmonella* Rubislaw gastroenteritis in humans was linked to the terrarium of a pet lizard [32].

In the current study, the isolation rate for *Salmonella* was much higher in the rainy than in the dry season. This finding is similar to reports from studies conducted in Uganda [33] and Southern Ethiopia [34] that investigated the frequency of *Salmonella* in different environmental samples collected during the rainy and dry seasons. In the rainy season, there is increased availability of contaminated water sources and potential flooding, which create favorable conditions for bacterial growth and survival. Also, in most areas in Africa, the spread of *Salmonella* is aided by inadequate sanitation and poor drainage systems [35]. In contrast, temperate climates record high occurrences of *Salmonella* during the summer months [36] because during warm temperatures, delayed refrigeration of food products creates ideal conditions for *Salmonella* to grow.

This study recorded higher antimicrobial resistance in commercial farms than in smallholder farms. This is not surprising because a recent study in the same area reported higher usage of antimicrobials in commercial farms than on the smallholder farm level [15]. Also, a significant level of multiple antibiotic resistance, especially resistance to fluoroquinolones, was observed. This is particularly concerning because fluoroquinolones are amongst the most important drugs for treating a wide range of infections in the country. Our study did not detect MDR; only one isolate was resistant to chloramphenicol. Contrary, other earlier studies done in Ghana have reported a high rate of MDR *Salmonella* in humans [11] and poultry [9]. But in agreement with the current finding, recent similar studies conducted in Nairobi and Ghana have recorded 100% [37] and 91% [20] susceptibility to chloramphenicol, respectively. This high *Salmonella* susceptibility to chloramphenicol is reassuring since it is commonly used in low-income countries to treat Salmonellosis [38]. Nonetheless, high resistance was observed for ampicillin and trimethoprim-sulfamethoxazole, which are likewise recommended for treating salmonellosis [38]. The high trimethoprim-sulfamethoxazole resistance observed probably reflects farmers' high use of sulfonamides in animal farming [39].

There were a few limitations to this study. Sampling was done in only two districts, so our results might not represent other geographical regions of Ghana. Dust and fecal samples were collected from only poultry farms, while soil samples were collected from poultry and livestock farms that raised pigs, goats, sheep, or cattle. Hence, caution must be taken when comparing dust and fecal isolates to isolates from the soil. Also, the *Salmonella* isolated from dust and soil may not be coming from the farm animals alone since feces from reptiles, rodents, and other non-farm animals may be part of the dust and soil collected from commercial and smallholder farms. Despite the aforementioned limitations, our study provides enhanced insights into the types of *Salmonella* serovars found in dust, soil, and animal feces from commercial and smallholder farms.

## Conclusion

In conclusion, this study reports on diverse *Salmonella* serovars circulating in environmental samples (dust, soil and feces) from commercial and smallholder farms in Ghana. The data shows that ecological niches might present a transmission reservoir for antibiotic-resistant *Salmonella*. Hence, it is important to monitor such niches for surveillance purposes, especially commercial farms during the rainy seasons, to enable the implementation of control strategies. Last but not least, these findings warrant encouraging good husbandry practices, such as farms having concrete floors and periodic dust removal from environments.

## Methods

### Study site and sample collection

**Fig. 2:**
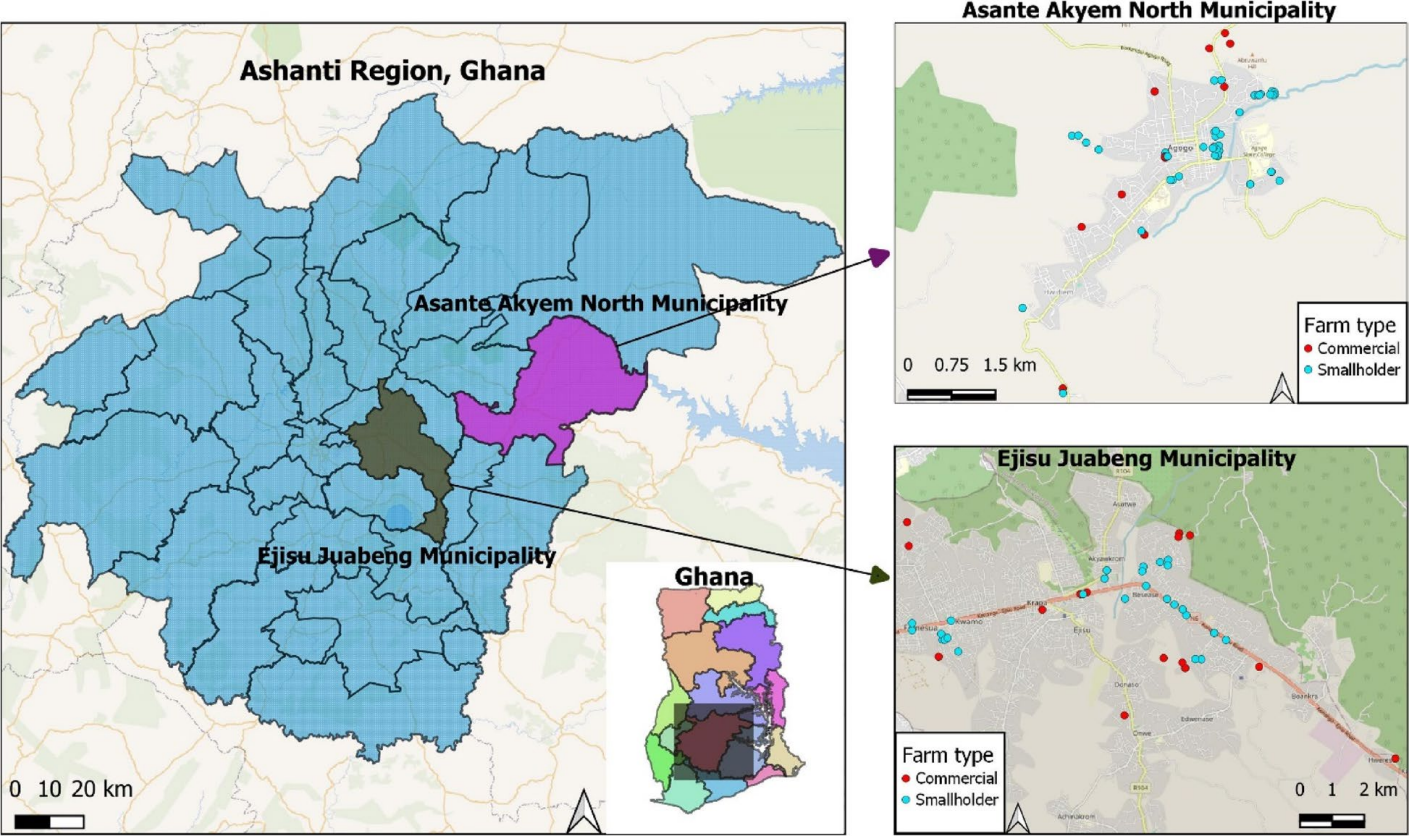
Location of commercial and smallholder farms sampled in the Asante Akyem North Municipality and the Ejisu Juabeng Municipality of Ghana. (The authors created this map using QGIS software)

This study was carried out in the Asante Akyem North Municipality, a rural community and the Ejisu Juabeng Municipality, a semi-urban community located in the Ashanti region of Ghana (Fig. 2). Ghana has a tropical climate with two main seasons. The rainy season lasts from April to October, and the dry season lasts from November to March. Sampling was done between April 2019 and November 2020. Commercial and smallholder farms were selected using snowball sampling. Following the farmers' consent to the study, soil, dust and pooled fecal samples were collected from the farm environments. Soil samples around the animal pen and farm environments were collected from poultry and livestock (pigs, sheep, goats and cattle) farms.

In contrast, dust and fecal samples were collected from poultry farms only. 1490 samples were collected from 30 commercial and 64 smallholder farms. Approximately 53.2% (n/N = 793/1490) of the samples were from commercial farms, and 46.8% (n/N = 697/1490) were from smallholder farms. For the environmental soil samples, 630 were collected from poultry farms, while 67, 67, 18, and 17 were collected from farms that kept pigs, sheep, goats, and cattle. Additionally, 409 pooled poultry fecal samples were collected, and 281 dust samples were obtained from poultry farms. A farm was considered commercial if it had at least 500 caged poultry and/or any quantity of caged livestock with an intensive housing system. In contrast, smallholder farms (small-scale agriculture) were households with free-roaming poultry (mainly indigenous breeds) and/or livestock with shelter provided by basic or temporary roofing. Farms with multiple pen houses were visited more than once; however, each was sampled only once.

Soil samples were collected from commercial and smallholder farms at 0-5 cm depth, with a core sampler measuring 5 cm in diameter and length. From each farm, two categories of soil samples were taken; soil from within ≤ 5 m around the pen structure and more than 5 m away from the pen structure but within the farm environment (including the working area, walkways, and part of the household where free-range animals roam and humans reside). Pooled fecal samples were collected from poultry farms using a pair of socks (strips mobs nurse cap, Hubei Zhiyue Non-woven products Co. Ltd) soaked in 0.9% normal saline. Socks were worn over a farm boot and used to take ten steps in a figure-of-eight-like pattern around the pen perimeter, as described by Andoh et al. [9]. Dust samples were collected from each poultry pen house using sterile socks moistened with 0.9% normal saline. The socks were used to clean the farms' fences, doors, and feeding/water troughs. All samples were placed in labelled sterile plastic containers and transported in a cool box (4–8 °C) to the Kumasi Center for Collaborative Research (KCCR) within 1–4 h for further processing.

### *Salmonella* culture and identification

Fecal, dust and soil (5 g) samples were pre-enriched in buffered peptone water (Oxiod) and incubated at 35–37℃ for 18–24 h in a normal atmosphere. This was further enriched in selenite broth (Difco, BD) and then incubated at 35–37℃ for 18–24 h in a normal atmosphere. The enrichment broth was then cultured on Xylose Lysine Deoxycholate agar (XLD) (Difco, BD) and incubated at 35–37℃ for 18–24 h in a normal atmosphere. Suspected *Salmonella* colonies were presumptively identified using a *Salmonella* latex test (Oxoid) and the analytical profile index test (API 20E, bioMérieux, Marcy l’Etoile, France). All *Salmonella* were confirmed using the automated VITEK 2 System and serotyped following the White-Kaufmann Le Minor scheme [40] at the National Reference Centre for *Salmonella* and Other Bacterial Enteric Pathogens at the Robert Koch Institute (RKI), Germany.

### Antimicrobial susceptibility testing

Antimicrobial susceptibility testing was performed using the Kirby Bauer disk diffusion method and interpreted following the European Committee on Antimicrobial Susceptibility Testing (EUCAST, version 12.0) guidelines. *Salmonella* Typhimurium ATCC 14028 was used as a reference strain for quality control. Confirmed *Salmonella* strains were tested against ampicillin, cefotaxime, cefoxitin, ceftazidime, chloramphenicol, ciprofloxacin, meropenem and trimethoprim-sulfamethoxazole. Isolates resistant to chloramphenicol, ampicillin, and trimethoprim-sulfamethoxazole were considered MDR [41]. Multiple antibiotic resistance was defined as resistance to three or more antimicrobials of different substance classes.

### Statistical analysis

Categorical variables were described using absolute frequencies and their corresponding percentages. Association between two categorical variables were shown using prevalence ratios and their corresponding 95% confidence intervals (CI). Crude and adjusted prevalence ratios (PR) and their respective 95% CIs were calculated in bivariate and multivariate analyses, respectively, using Poisson regression to show possible factors associated with *Salmonella* frequency. Because of the exploratory nature of this study, *p*-values were not calculated. All analyses were conducted using R statistical software (version 4.1.1), and the *epiR* (2.0.19) package was applied to calculate PRs. The ggplot2 package was used to plot bar charts. QGIS software, version 3.24.0 [42], was used to draw a map showing the sampled farms' geographical location.

## Data Availability

The data used to support the findings of this study are available from the corresponding author upon request.

## Abbreviations

AMR: Antimicrobial resistance
EUCAST: European Committee on Antimicrobial Susceptibility Testing
MDR: Multidrug-resistant
API: Analytical profile index test
KCCR: Kumasi Center for Collaborative Research
XLD: Xylose Lysine Deoxycholate agar
